# Associations between Psychological Problems and Quality of Life in Pediatric Short Stature from Patients’ and Parents’ Perspectives

**DOI:** 10.1371/journal.pone.0153953

**Published:** 2016-04-20

**Authors:** Julia Hannah Quitmann, Monika Bullinger, Rachel Sommer, Anja Christine Rohenkohl, Neuza Maria Bernardino Da Silva

**Affiliations:** 1 University Hamburg-Eppendorf, Department of Medical Psychology, Hamburg, Germany; 2 University of Coimbra, Cognitive and Behavioral Center for Research and Intervention, Coimbra, Portugal; Harbin Medical University, CHINA

## Abstract

Short stature has been associated with psychosocial impairments, but whether treatments and achieved height impact on health-related quality of life (HrQoL) and psychological functioning of children/adolescents is still controversial. This study aimed to examine the effects of height deviation and treatment status on psychosocial adaptation outcomes and to identify clinical and psychosocial determinants of internalizing/externalizing problems in a large cohort of short statured children/adolescents from seven European countries. Participants were 345 children aged 8–18 years with a clinical diagnosis of short stature and 421 parents of 4–18 year-old patients. Children and parents reported on psychological problems (Strengths and Difficulties Questionnaire), generic (KIDSCREEN) and condition-specific HrQoL (QoLISSY). According to analyses of covariance, children/adolescents with current short stature presented more parent-reported internalizing problems and lower self- and parent-reported condition-specific HrQoL, compared to patients with an achieved height above -2SD. Treated children self-reported better HrQoL than the untreated group. Hierarchical regression analysis showed that, rather than height–related clinical variables, children’s sex, younger age and poorer HrQoL were the best predictors of psychological problems, explaining 39% of the variance in patient- and 42% in parent-reported internalizing problems, and 22% of the variance in patient- and 24% in parent-reported externalizing problems. Treatment status also moderated the negative links between patient-reported HrQoL and internalizing problems, explaining 2% of additional variance. These results suggest that children with current short stature are at greater risk for internalizing problems. Routine assessment of HrQoL in pediatric healthcare may help identify children for referral to specialized psychological assessment and intervention.

## Introduction

Evidence-based research in pediatrics has demonstrated that while children facing a chronic health condition are at risk for psychosocial impairments, patients with short stature rarely present with clinically relevant behavioral problems [1, 2]. To better understand psychological functioning, the role of adaptation and the potential impact of statural height and growth hormone (GH) treatment effect need to be studied [3]. This paper intends to contribute to the scientific debate about the psychosocial adaptation of children with short stature by conceptualizing internalizing and externalizing problems as outcome criteria, and examining the role of clinically reported height and treatment status, as well as patient- and parent-reported health-related quality of life (HrQoL) as factors explaining outcome variance in a large European sample of short statured patients.

### Short Stature and Growth Hormone Treatment

Short stature is defined as a height of 2.0 or more standard deviations (SD) below the population-specific mean height for age and gender [4]. Representing approximately 3% of the population, short stature has been associated with over 400 genetic and endocrine diseases, as well as with environmental and psychosocial factors including socio-economic conditions, malnutrition, psychosocial distress and emotional deprivation [5, 6]. Growth problems in childhood are a frequent reason for referral to pediatric endocrinologists with the most common endocrine form of short stature being growth hormone deficiency (GHD) [7, 8]. Children and adolescents with GHD have a complete or partial absence of GH secretion or lower levels of growth factors such as the insulin like growth factor (IGF-I) [9, 10]. However, 60–80% of children with short stature have sufficient GH secretion, normal birth size and no evidence of systemic disease, psychiatric disorders or malnutrition [11, 12]. This heterogeneous group is classified as idiopathic short stature (ISS) [7, 13].

Strong evidence accumulated for the clinical effectiveness of recombinant human GH (rhGH) treatment via daily subcutaneous injection to increase growth velocity and to normalize adult height in children with GHD [14]. Studies have also supported the effectiveness of rhGH treatment to improve final height in children with ISS, based on the premise that normal levels of GH secretion in these patients may be insufficient to stimulate GH receptors [15, 16]. However, rhGH therapy is approved by the European Medicines Agency to stimulate growth only in patients with GHD, not with ISS [17]. If catch-up growth is slow or GH treatment is unsuccessful, children may remain short compared to peers resulting significant psychosocial impairments [15].

### Psychosocial Adaptation Outcomes in Pediatric Short Stature

Alongside with environmental barriers to autonomy development, short statured children/adolescents tend to be underestimated by peers, teachers and parents, are frequently teased, rejected or overprotected and, thus, are more likely to experience isolation and discrimination [18, 19]. Young patients with short stature were reported to be at increased risk for psychosocial distress due to stigmatization, bullying, social isolation, juvenalization and low self-esteem [20]. Older studies suggests that short statured children encounter significant social, academic and psychological difficulties due to their condition [21] and experience more internalizing problems such as social isolation and lack of appropriate aggressive drive than normal height children [22, 23]. Studies examining the behavior profile of short statured children reported higher levels of behavioral problems, compared to a control sample of normal-statured children [24] and a representative population sample [25]. Generally, clinically diagnosed short-statured children were found to report lower HrQoL than population-based normal-statured reference groups [26, 27].

Despite findings that short statured patients have more physical and psychosocial impairments than their normal-height peers, controversies still exist regarding the clinical relevance of those findings [3]. In a systematic review, Wheeler, Bresnahan, Shephard, Lau, and Balk (2004) concluded that children with short stature tend to score lower than their peers on functional tests but few patients scored outside the normal range [28]. A growing set of recent studies support the hypothesis that short statured children may adapt well to their height, despite of psychosocial disadvantages. For instance, a population-based American study found only marginally higher levels of self-perceived peer victimization for shorter children and no differences in a range of social, emotional and behavioral outcomes, compared to average height children [29]. A recent Korean study found lower general health perceptions in shorter patients but no significant differences in psychosocial burden between height groups [4]. Also the Wessex Growth Study found no evidence that childhood short stature per se significantly impacted on the psychosocial functioning in early adulthood [30].

### Do Height and GH Treatment matter?

Population-based studies have suggested that height has only a negligible impact on psychosocial adaptation, challenging the proclaimed association between short stature and poor HrQoL, both in adults [31] and adolescents [32]. In a review of psychosocial consequences of short stature, Sandberg and Gardner (2015) concluded that, despite the variable occurrence of negative social experiences, short stature as an isolated physical characteristic is not associated per se with psychological maladaptation [3]. Other studies showed that, rather than height, male gender, lower intelligence, the presence of a younger but taller sibling, being treated as younger than chronological age and low family socio-economic status are best predictors of children’s psychosocial adaptation [33–35].

Improving HrQoL and psychological health through an increase in height has been considered a major goal of rhGH treatment, but studies yielded inconsistent findings [7, 36]. For example, Sheppard and colleagues (2006) found that HrQoL improved in GHD patients after 6 months of GH treatment, albeit statistically insignificant [37]. Increases in HrQoL and self-esteem in children with ISS receiving GH treatment were reported by physicians, but not by the children or their parents [38]. Other studies did not find rhGH induced height gain to improve HrQoL and psychological functioning in short statured children (e.g., [39, 40]).

### Rationale and Objectives

As outlined above, HrQoL and psychological problems are two indicators, of psychosocial adaptation in children/adolescents with short stature. Internalizing and externalizing problems have traditionally been recommended as specific psychological functioning outcomes because of their high prevalence in the population and in children with chronic conditions or disabilities [41]. More recently HrQoL has been introduced as a multidimensional health indicator, covering the physical, emotional and social aspects of well-being and functioning [42]. Assessing psychological problems and HrQoL as psychosocial outcomes allows an extension beyond the psychopathological conceptualization of child mental health and the representation of adaptation as a process that accounted for resiliency and variability on specific indicators [43].

Short stature is expected to impact on both indicators of psychosocial adaptation; however most studies have examined HrQoL and psychological problems separately, and little is known about the associations between them. Therefore, we introduce HrQoL assessment as a screening tool to detect “hidden” psychosocial and functional morbidities from the patients’ and parents’ perspectives in pediatric clinical practice [44]. Accordingly, our study aimed at: (1) describing the patient- and parent-reports of HrQoL and psychological problems, based on height deviation and treatment groups; (2) examining the main and interaction effects of clinical variables, HrQoL and psychosocial determinants for explaining the variance of internalizing and externalizing problems in European short statured children/adolescents and (3) providing a better understanding of the psychosocial consequences of GHD and ISS in order to help clinicians to identify vulnerable groups of pediatric patients.

## Methods

### Participants and Procedures

Data originated from the Quality of Life in Short Stature Youth (QoLISSY) project, which developed and validated a cross-culturally disease-specific HrQoL measure for clinically referred 8–18 year old short statured patients, and for parents of these children aged 4–18. It was simultaneously conducted in five European countries using (1) focus-groups with item generation; (2) pilot test with cognitive debriefing; and (3) field test with re-test [27]. Over time, the QoLISSY questionnaire was sequentially translated and validated in Belgium and the Netherlands [45–47] and further validations have just been completed (US) [45].

The data used in this analysis include subjects participating in the original 5-country development and testing of the QoLISSY plus two follow-on validation studies conducted in Belgium and The Netherlands. Subjects were recruited in pediatric endocrine centers of the seven European countries (Sweden, Germany, France, UK, Spain, Belgium and The Netherlands), upon approval by the respective Ethic Committees (namely, die Ethikkommission der Ärztekammer Hamburg, Deutschland; the METC; Onderwijscoordinatie & projecten in 1201 DA Hilversum; the Regionala etikprövningsnämnden I Göteburg, the Universitair Ziekenhuis Brussel, Commissie Medische Ethiek, the Comité d’ethique de Toulouse, France; the research ethics committee, NHS Lothian, Edinburgh, and the CEIC Hospital Clínic de Barcelona). The study was performed following data protection requirements of the European Parliament (Directive 95/46/EC of the European Parliament and of the Council of 24 October 1995 on the protection of individuals with regard to the processing and free movement of personal data).

Patient inclusion criteria were (1) a previous clinical diagnosis of GHD or ISS; (2) age between 8 and 18 years; (3) height deviation more than -2SD below age- and gender-adjusted population norms at the time of diagnosis; (4) absence of comorbidities with severe mental disorders and/or other chronic health conditions; and (5) cognitive ability to understand and complete the questionnaires.

In compliance with the APA ethical principles regarding research with human participants [48], detailed information about the study aims and procedures was provided in the respective language when families attended the clinical centers for regular appointments. Informed consents were obtained from parents, together with informal assents from children/adolescents, as well as permission to extract medical data from clinical records through their physicians. The field test questionnaires, to be independently completed by patients and parents, were given to the families when they visited the clinical centers or were sent by mail, together with a pre-stamped envelope for returning the completed questionnaires. All questionnaires were provided to subjects in their local language.

Anonymized data were entered into a project specific SPSS database, which included the computation of height deviation at the time of enrollment in the study with reference to the national norms for age and gender. Overall, 345 patients aged between 8 and 18 years and 421 parents of children aged between 4 and 18 years were included. The socio-demographic and clinical characteristics of the sample are presented in Table 1.

**Table 1:** Socio-demographic and clinical characteristics of the sample.

		Children 4–7	Children 8–12	Adolescents 13	Parents
		years-old	years-old	18 years-old	
		(*n* = 76)	(*n* = 159)	(*n* = 186)	(*n* = 421)
**Sociodemographic characteristics**					
Age, *M* (*SD*)		6.06 (1.03)	10.28 (1.46)	14.82 (1.48)	43.77 (17.92)
Sex, *n* (%)	Male	44 (57.9%)	86 (54.1%)	117 (62.9%)	46 (10.9%)
	Female	32 (42.1%)	73 (45.9%)	69 (37–1%)	309 (73.4%)
	*Missing*	-	-	-	66 (15.7%)
Country, *n* (%)	Sweden	3 (3.9%)	14 (8.8%)	59 (31.7%)	76 (18.1%)
	Germany	24 (31.6%)	41 (25.8%)	26 (14.0%)	91 (21.6%)
	France	11 (14.5%)	30 (18.9%)	23 (12.4%)	64 (15.2%)
	UK	3 (3.9%)	16 (10.1%)	16 (8.6%)	35 (8.3%)
	Spain	15 (19.7%)	27 (17.0%)	17 (9.1%)	59 (14.0%)
	Belgium	12 (15.8%)	8 (5.0%)	19 (10.2%)	39 (9.3%)
	The Netherlands	8 (10.5%)	23 (14.5%)	26 (14.0%)	57 (13.5%)
**Clinical characteristics**					
Diagnosis, *n* (%)	GHD	28 (36.8%)	47 (29.6%)	77 (41.4%)	
	ISS	48 (63.2%)	112 (70.4%)	109 (58.6%)	
Treatment status, *n* (%)	GH treatment	24 (31.6%)	71 (44.7%)	98 (52.7%)	
	Untreated	52 (68.4%)	88 (55.3%)	88 (47.3%)	
Current height deviation, *n* (%)	Above –2 SD (achieved normal height)	27 (35.5%)	72 (45.3%)	92 (49.5%)	
	Below –2 SD (current short stature)	45 (59.2%)	84 (52.8%)	91 (48.9%)	
	*Missing*	4 (5.3%)	3 (1.9%)	3 (1.6%)	
Patient-reported psychological problems, *n* (%)	Normal	-	119 (74.8%)	142 (76.3%)	
	Borderline	-	17 (10.7%)	11 (5.9%)	
	Abnormal	-	11 (6.9%)	12 (6.5%)	
	*Missing*	-	12 (7.5%)	21 (11.3%)	
Parent-reported psychological problems, *n* (%)	Normal	41 (53.9%)	109 (68.6%)	138 (74.2%)	
	Borderline	10 (13.2%)	16 (10.1%)	16 (8.6%)	
	Abnormal	22 (28.9%)	31 (19.5%)	28 (15.1%)	
	*Missing*	3 (3.9%)	3 (1.9%)	4 (2.2%)	

### Instruments

#### The Strengths and Difficulties Questionnaire (SDQ)

The patients’ psychological problems were measured using the self- and parent-rated SDQ [49, 50], a brief questionnaire developed with reference to the Diagnostic and Statistical Manual of Mental Disorders (DSM-IV; [51]). The questionnaire comprises five scales with five items each: Emotional Symptoms, Conduct Problems, Hyperactivity/Inattention, Peer Relationship Problems and Prosocial Behavior. The items are to be answered using a Likert-type response scale with three options (0 = *not true*, 1 = *somewhat true* and 2 = *certainly true*), providing mean scores for each scale. Apart from the Prosocial Behavior scale (which was not used due to its inadequate reliability of α = .66 for patient-reports and α = .64 for parent-reports), the remaining 20 items were coded into Internalizing and Externalizing Problems, following recent recommendations for assessing low-risk or general population samples [52]. A Total Difficulties score was also computed, with higher scores indicating more psychological problems. The SDQ has been adapted and translated into more than 30 languages [53] and is suitable for cross-cultural research. In the current sample, both self- and parent-rated scales presented good reliability (Table 2).

**Table 2:** Descriptive statistics and univariate analyses of covariance by height deviation and treatment groups.

	Scale reliability	GH treatment		Untreated		Treatment main effects		Height deviation main effects		Treatment X height deviation interaction effects	
		Achieved normal height	Current short stature	Achieved normal height	Current short stature						
**Patient-reports**		***n* = 103**	***n* = 49**	***n* = 52**	***n* = 104**						
	α	*M* (*SD*)	*M* (*SD*)	*M* (*SD*)	*M* (*SD*)	*F*	*p*	*F*	*p*	*F*	*p*
1. Internalizing problems	.72	0.39 (0.30)	0.47 (0.35)	0.36 (0.33)	0.45 (0.36)	0.24	.62	2.72	.10	0.01	.91
2. Externalizing problems	.76	0.55 (0.36)	0.62 (0.36)	0.57 (0.38)	0.58 (0.34)	0.82	.37	0.81	.37	0.57	.45
3. Generic QoL	.94	79.43 (11.29)	78.56 (11.10)	72.23 (12.50)	75.25 (11.72)	16.72	< .01	0.02	.90	3.23	.07
4. Specific QoL	.94	84.86 (12.16)	58.76 (25.29)	77.45 (18.08)	59.37 (21.89)	0.37	.54	51.13	< .01	0.51	.48
5. Coping	.83	51.59 (26.24)	56.56 (19.25)	37.00 (22.00)	56.74 (19.76)	1.30	.26	17.82	< .01	4.15	.04
6. Beliefs	.86	78.60 (26.52)	51.49 (31.08)	74.44 (25.38)	64.29 (27.67)	9.15	< .01	18.89	< .01	2.00	.16
**Parent-reports**		***n* = 116**	***n* = 73**	***n* = 75**	***n* = 147**						
	α	*M* (*SD*)	*M* (*SD*)	*M* (*SD*)	*M* (*SD*)	*F*	*p*	*F*	*p*	*F*	*p*
1. Internalizing problems	.74	0.38 (0.36)	0.54 (0.40)	0.32 (0.27)	0.44 (0.37)	3.48	.06	8.51	< .01	0.25	.62
2. Externalizing problems	.81	0.58 (0.47)	0.67 (0.36)	0.49 (0.43)	0.54 (0.34)	2.40	.12	0.89	.35	0.40	.53
3. Generic QoL	.94	75.75 (10.86)	74.45 (11.50)	70.60 (10.84)	72.70 (10.18)	3.25	.07	0.05	.82	1.24	.27
4. Specific QoL	.94	83.19 (16.08)	62.31 (22.10)	74.81 (18.60)	64.05 (20.43)	0.80	.37	30.94	< .01	2.52	.11
5. Coping	.82	41.19 (25.87)	48.97 (15.21)	38.97 (19.77)	48.64 (19.15)	0.21	.64	9.74	< .01	0.08	.78
6. Beliefs	.86	76.25 (25.49)	59.38 (27.55)	78.98 (23.74)	67.47 (25.80)	3.17	.08	15.57	< .01	0.27	.61
7. Future	.86	87.44 (18.70)	67.26 (14.55)	84.09 (18.78)	69.75 (26.30)	1.60	.21	29.13	< .01	0.14	.71
8. Effect on parents	.89	76.47 (22.27)	61.39 (21.94)	76.22 (22.34)	65.57 (23.81)	3.99	.05	13.75	< .01	0.11	.75

#### The KIDSCREEN-52

The KIDSCREEN-52 (The KIDSCREEN Group Europe, 2006) is a generic instrument to assess HrQoL on the population level in children/adolescents between 8 and 18 years of age [54]. The questionnaire consists of 52 items, clustered into 10 domains: Physical Well-being, Psychological Well-being, Moods and Emotions, Self-Perception, Autonomy, Parent Relations and Home Life, Social Support and Peers, School Environment, Social Acceptance/Bullying, and Financial Resources. The items are to be answered on a 5-point Likert scale ranging from 1 (*never*/*not at all*) to 5 (*always*/*extremely*), providing standardized scores (ranging from 0 to 100) for each domain and total score, where higher values indicate better HrQoL. Due to its extensive cross-cultural development process, the KIDSCREEN-52 is a valuable tool for generic HrQoL assessment across countries and languages. Patient-report and parent-report form, demonstrated good reliability in the current sample (see Table 2).

#### The QoLISSY Questionnaire

The QoLISSY questionnaire is a condition-specific instrument targeting patient-reported HrQoL of 8–18 year-old children/adolescents with short stature, as well as the parent-reported HrQoL of 4–18 year-old patients [55]. It consists of a core module with 22 items, assessing three HrQoL domains: Physical, Social and Emotional. In addition, three scales assessing psychosocial determinants of pediatric HrQoL are provided: Coping, Height-related Beliefs and Treatment Experiences. The parent-report version includes two additional scales assessing Concerns about the Future and the Effects on Parents. All items were answered using a standard 5-point Likert scale ranging from 1 (*not at all*/*never*) to 5 (*extremely*/*always*), providing standardized scores (0–100) for each scale and for a total HrQoL score, representing the mean of the three core domains standardized scores, with higher values indicating better HrQoL. Because we included both GH treated and untreated patients, the Treatment Experiences scale was excluded from the analyses. The Cronbach’s alpha values for each patient- and parent-reported scale and HrQoL total score are presented in Table 2.

#### Socio-demographic and Clinical Data

The socio-demographic data included patients’ sex, date of birth and nationality. Physician reported clinical data included diagnosis (GHD or ISS), treatment status and height at time of diagnosis and at time of assessment. The treated group consisted of patients receiving rhGH treatment at the time of assessment and before; the untreated group had never been treated. Although a height deviation greater than -2SD at time of diagnosis was required for patient inclusion, some had an achieved height above -2SD at the time of assessment due to treatment. Thus, the current height deviation was categorized into two groups: achieved normal height (height deviation > -2 SD) and current short stature (height deviation ≤ -2 SD).

### Data Analysis

Statistical analyses were conducted with SPSS v.20.0 (SPSS Inc., Chicago, IL). Except for sociodemographic and clinical variables, missing data at random with less than 25% unendorsed items per domain were replaced with the respective individual mean score. Descriptive statistics were calculated for all variables including patient- and parent-reported SDQ scores, which were categorized based on published cut-off values to differentiate between normal, borderline and abnormal scores [49]. Differences between current height deviation (achieved normal height *vs*. current short stature) and treatment groups (GH treated *vs*. untreated) on children’s psychological problems (i.e., internalizing and externalizing problems), HrQoL (generic and condition-specific), and psychosocial determinants (i.e., coping, height-related beliefs, concerns about the future and effects on parents) were tested with two-way multivariate analyses of covariance (MANCOVA), controlling for child’s age, sex and diagnosis. When multivariate effects were significant, univariate analyses were performed to examine significant differences between groups.

Pearson’s correlation coefficients between the main clinical and socio-demographic variables and children’s psychological problems, generic and condition-specific HrQoL and psychosocial determinants were computed to select the appropriate covariates and interaction terms for introduction into the regression model [56]. After the inclusion of covariates into the first step of the regression equation, the clinical variables, HrQoL and its psychosocial determinants, and the interaction terms were subsequently entered as patient- or parent reported predictors of internalizing and externalizing problems [57]. Continuous independent variables were mean centered to reduce multicollinearity with the interaction terms and increase interpretability of regression coefficients [57, 58]. According to the guidelines for testing multiple moderating effects [59], interaction terms were introduced conjointly in the same step of the regression equation. Effect sizes of main and interaction effects were based on *R*^2^ values, considering *R*^2^ ≥ .02 as small, *R*^2^ ≥ .13 as medium and *R*^2^ ≥ .26 as large effects [60]. Using the Modgraph computational tool [61], the significant interaction effects were plotted from the estimates of the dependent variable at different values of the moderator, and the strength and significance of each regression line was analyzed with post-hoc simple slope computations.

## Results

### Descriptive Statistics and Analyses of Covariance

Based on the SDQ cut-off values [49], 83.6% of patient- and 70.1% of parent-reports were within the normal range, while 8.8% of children and 10.2% of parents scored within the borderline range, and 7.6% of children and 19.7% of parents reported clinically significant psychological problems (Table 1). The prevalence of patient-reported psychological problems is comparable to population norms from UK [62], Germany [63] and Sweden [64]. However, twice as many children were assessed within the abnormal range by their parents, according to aforementioned norms, especially in younger age groups. No multivariate main or interaction effects of height deviation or treatment status were found on patient-reported psychological problems, but a significant multivariate main effect of height deviation was found on parent-reports, Wilks’ Lambda = .97, *F*_(2,295)_ = 4.34, *p* = .01, ŋ_p_^2^ = .03. The univariate analyses showed that parents of children with current short stature reported significantly more internalizing problems than parents of children with achieved normal height (Table 2).

HrQoL analyses yielded significant multivariate main effect of treatment status, Wilks’ Lambda = .93, *F*_(2,238)_ = 9.26, *p* < .01, ŋ_p_^2^ = .07, and current height deviation, Wilks’ Lambda = .82, *F*_(2,238)_ = 26.50, *p* < .01, ŋ_p_^2^ = .18, on child-reports, and a significant multivariate effect of current height deviation on parent-reports, Wilks’ Lambda = .87, *F*_(2,236)_ = 17.51, *p* < .01, ŋ_p_^2^ = .13. As depicted in Table 2, treated patients reported better generic HrQoL, while patients with achieved normal height presented better self- and parent-reported condition-specific HrQoL. In addition, significant multivariate main effects of treatment status, Wilks’ Lambda = .96, *F*_(2, 241)_ = 5.15, *p* < .01, ŋ_p_^2^ = .04, and current height deviation, Wilks’ Lambda = .87, *F*_(2, 241)_ = 17.98, *p* < .01, ŋ_p_^2^ = .13, as well as a significant multivariate interaction effect between the two factors, Wilks’ Lambda = .98, *F*_(2, 241)_ = 3.11, *p* = .05, ŋ_p_^2^ = .03, were found on children’s psychosocial determinants. Here, untreated children with current short stature presented more adaptive better coping, *F*_(1,122)_ = 25.24, *p* < .01, and less adaptive height-related beliefs, *F*_(1,124)_ = 3.92, *p* = .05, than untreated children with an achieved height above -2SD; while GH treated patients with achieved normal height reported more adaptive health-related beliefs, *F*_(1,131)_ = 14.95, *p* < .01, but similar coping, *F*_(1,128)_ = 2.21, *p* = .14, compared to treated patients with current short stature. Finally, a significant multivariate main effect of current height deviation was also found on parents’ psychosocial variables, Wilks’ Lambda = .87, *F*_(4, 257)_ = 9.81, *p* < .01, ŋ_p_^2^ = .13. Parents of children with current short stature reported better coping but lower scores on height-related beliefs, concerns about the child’s future and effects of the child’s short stature on parents, compared to the group with achieved normal height.

### Preliminary Correlation Analyses

Weak to moderate negative associations exist between psychological problems and HrQoL, that is, better quality of life was associated with fewer psychological problems, in both patient- and parent-reported samples (Table 3). Patient-reported psychological problems were negatively associated with height-related beliefs, and parent-reported psychological problems were negatively correlated with coping, height-related beliefs, concerns about the child’s future and effects of the child’s short stature on parents. Current or prior GH treatment was associated with better patient-reported generic and condition-specific HrQoL, and with higher scores in parent-reported specific HrQoL and fewer (better scores on the domain) concerns about the future. Current short stature was correlated with higher patient-reported coping efforts and less adaptive height-related beliefs, as well as with parent-reported reduced condition-specific HrQoL, better coping and lower scores in beliefs, concerns about the future and effects on parents. Coping can be seen as an effort to adapt to difficult conditions and increased coping efforts are expected when patients’ perceived health is compromised [65]. The significant associations between clinical and psychosocial variables were introduced into the regression equations as interaction terms. Female sex was associated with more patient-rated internalizing problems and fewer externalizing problems from patient and parent perspectives; and younger age was related to more internalizing problems in parent-report and more externalizing problems patient- and parent-reports. Therefore, children’s sex and age were included as covariates, when appropriate.

**Table 3:** Matrix of inter-correlations among behavioral problems, HrQoL, psychosocial determinants and socio-demographic and clinical variables.

	Behavioral problems		HrQoL		Psychosocial determinants			Socio-demographic and clinical variables				
**Patient-reports**	1	2	3	4	5			Sex ^a^	Age	Diagnosis ^b^	Treatment status ^c^	Height deviation ^d^
1. Internalizing problems	-							-.13^*^	-.10	.01	.01	.11
2. Externalizing problems	.36^**^	-						.20^**^	-.16^*^	< .01	.02	.08
3. Generic QoL	-.51^**^	-.27^**^	-					.02	-.05	< .01	-.15^**^	-.07
4. Specific QoL	-.52^**^	-.25^**^	.26^**^	-				.05	.06	-.27^**^	-.21^**^	-.41^**^
5. Coping	-.07	-.09	.31^**^	-.06	-			.02	-.13^*^	-.02	-.01	.16^**^
6. Beliefs	-.39^**^	-.20^**^	.22^**^	.70^**^	-.02			-.12^*^	.03	-.20^**^	-.02	-.20^**^
**Parent-reports**	1	2	3	4	5	6	7	Sex ^a^	Age	Diagnosis ^b^	Treatment status ^c^	Height deviation ^d^
1. Internalizing problems	-							< .01	-.19^**^	< .01	-.04	.16^**^
2. Externalizing problems	.46^**^	-						.16^**^	-.28^**^	-.07	-.08	.03
3. Generic QoL	-.52^**^	-.26^**^	-					-.08	-.04	-.08	-.08	-.10
4. Specific QoL	-.42^**^	-.15^**^	.41^**^	-				.08	.20^**^	-.24^**^	-.18^**^	-.37^**^
5. Coping	-.16^**^	-.05	.27^**^	-.01	-			-.06	-.01	.04	.04	.12^*^
6. Beliefs	-.38^**^	-.21^**^	.30^**^	.62^**^	< .01	-		-.08	.05	-.09	-.01	-.21^**^
7. Future	-.29^**^	-.11^*^	.25^**^	.72^**^	-.06	.68^**^	-	-.02	.03	-.21^**^	-.14^**^	-.29^**^
8. Effect on parents	-.44^**^	-.24^**^	.24^**^	.70^**^	< .01	.57^**^	.64^**^	-.03	.20^**^	-.16^**^	-.08	-.26^**^

^a^ Children’s sex: 0 –female, 1 –male

^b^ Diagnosis: 0 –GHD, 1 –ISS

^c^ Treatment status: 0 –treated; 1 –untreated

^d^ Current height deviation: 0 –achieved normal height (> -2sd), 1 –current short stature (≤ -2sd).

^*^ *p* ≤ .05

^**^ *p* ≤ .01, two-tailed

### Determinants of Internalizing Problems

Regression analyses showed main and interaction effects of clinical variables (i.e., treatment status and current height deviation), generic and condition-specific HrQoL and psychosocial variables (i.e., coping and height-related beliefs) on patient- and parent-reported internalizing problems (Tables 4 and 5).

**Table 4:** Main and interaction effects of clinical and psychosocial variables on patient-reported internalizing problems.

	Patient-reported internalizing problems							
	Covariates		Clinical variables main effects		Psychosocial variables main effects		Interaction effects	
	Δ*R*^2^ = .02		Δ*R*^2^ = .02		Δ*R*^2^ = .37		Δ*R*^2^ = .02	
	Δ*F*_(1, 291)_ = 6.14^*^		Δ*F*_(2, 289)_ = 2.34		Δ*F*_(4, 285)_ = 44.64^**^		Δ*F*_(4, 281)_ = 2.96^*^	
	β	*t*	β	*t*	β	*t*	β	*t*
Children’s sex ^a^	-.14	-2.48^*^	-.14	-2.46^*^	-.11	-2.31^*^	-.10	-2.15^*^
Treatment status ^b^			-.03	-0.48	-.14	-2.79^**^	-.13	-2.51^*^
Current height deviation ^c^			.13	2.16^*^	-.04	-0.79	-.04	-0.75
Generic QoL					-.34	-6.29^**^	-.49	-5.96^**^
Specific QoL					-.37	-5.04^**^	-.20	-1.98^*^
Coping					-.01	-0.06	-.02	-0.36
Beliefs					-.09	-1.40	-.17	-2.35^*^
Generic QoL * Treatment							.16	2.08^*^
Specific QoL * Treatment							-.21	-2.66^**^
Coping * Height deviation							.04	0.60
Beliefs * Height deviation							.12	1.88
Model summary	*R*^2^ = .02		*R*^2^ = .04		*R*^2^ = .41		*R*^2^ = .43	
	*F*_(1, 291)_ = 6.14^*^		*F*_(3, 289)_ = 3.63^*^		*F*_(7, 285)_ = 28.01^**^		*F*_(11, 281)_ = 19.39^**^	

^a^ Children’s sex: 0 –female, 1 –male

^b^ Treatment status: 0 –treated; 1 –untreated

^c^ Current height deviation: 0 –achieved normal height (> -2sd), 1 –current short stature (≤ -2sd).

^*^ *p* ≤ .05

^**^ *p* ≤ .01, two-tailed.

**Table 5:** Main and interaction effects of clinical and psychosocial variables on parent-reported internalizing problems.

	Parent-reported internalizing problems							
	Covariates		Clinical variables main effects		Psychosocial variables main effects		Interaction effects	
	Δ*R*^2^ = .01		Δ*R*^2^ = .01		Δ*R*^2^ = .42		Δ*R*^2^ = .02	
	Δ*F*_(1, 216)_ = 2.58		Δ*F*_(2, 214)_ = 1.18		Δ*F*_(6, 208)_ = 25.74^**^		Δ*F*_(7, 208)_ = 1.10	
	β	*t*	β	*t*	β	*t*	β	*t*
Children’s age	-.11	-1.60	-.10	-1.45	-.04	-0.68	-.05	-0.97
Treatment status ^a^			-.10	-1.33	-.14	-2.46^*^	-.11	-1.75
Current height deviation ^b^			.09	1.20	.01	0.08	-.003	-0.04
Generic QoL					-.44	-7.49^**^	-.42	-6.98^**^
Specific QoL					-.08	-0.80	.22	1.39
Coping					-.09	-1.64	-.05	-0.75
Beliefs					-.14	-1.82	-.22	-1.94^*^
Future					.11	1.26	.16	1.01
Effects on parents					-.30	-3.67^**^	-.44	-3.75^**^
Specific QoL * Treatment							-.07	-0.60
Future * Treatment							.09	0.65
Specific QoL * Height deviation							-.27	-2.02^*^
Coping * Height deviation							-.05	-0.71
Beliefs * Height deviation							.08	0.75
Future * Height deviation							-.10	-0.66
Effects on parents * Height deviation							.15	1.38
Model summary	*R*^2^ = .01		*R*^2^ = .02		*R*^2^ = .44		*R*^2^ = .46	
	*F*_(1, 216)_ = 2.58		*F*_(3, 214)_ = 1.65		*F*_(9, 208)_ = 18.09^**^		*F*_(16, 201)_ = 10.69^**^	

^a^ Treatment status: 0 –treated; 1 –untreated

^b^ Current height deviation: 0 –achieved normal height (> -2sd), 1 –current short stature (≤ -2sd).

^*^ *p* ≤ .05

^**^ *p* ≤ .01, two-tailed.

While controlling for children’s sex, a significant main effect of psychosocial variables was found, explaining 37% of the variance in patient-reported internalizing problems; lower generic and condition-specific HrQoL significantly accounted for more internalizing problems (Table 4). The insertion of the interaction terms between HrQoL and treatment status contributed to a significant increase of 2% of the explained variance in patient-rated internalizing problems.

**Fig 1:**
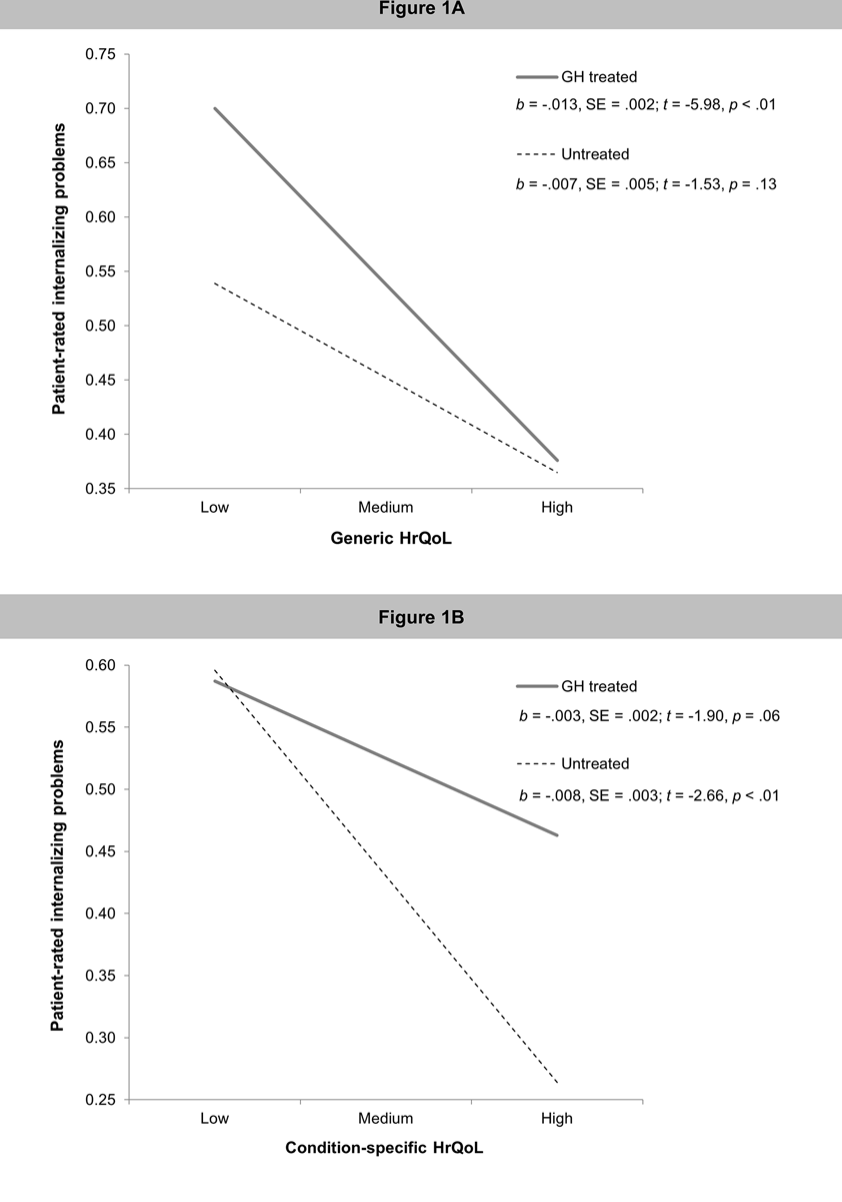
**The moderating effect of treatment status on the association between (1A) patient-reported generic HrQoL and internalizing problems; and (1B) patient-reported condition-specific HrQoL and internalizing problems.**

Post-hoc simple slope analyses (Fig 1A) revealed that higher levels of generic HrQoL were associated with less internalizing problems in GH treated, but not in untreated patients. Conversely, better condition-specific HrQoL was associated with fewer internalizing problems in untreated patients, but not in those receiving GH treatment (Fig 1B).

From the parents’ perspective (Table 5), lower scores in generic HrQoL and effects of the child’s short stature on parents explained a significant portion (42%) of the variance in internalizing problems. Although the interaction effect between condition-specific HrQoL and current height deviation reached statistical significance, its contribution to explain additional variance in parent-reported internalizing problems was non-significant.

### Determinants of Externalizing Problems

Tables 6 and 7 present the results from the regression analyses examining the main and interaction effects of clinical psychosocial and HrQoL variables on externalizing problems from patient and parent perspectives. Regarding patient-reports, significant main effects of socio-demographic and psychosocial variables on externalizing problems were present, explaining, 6% and 16% of its variance, respectively (Table 6). Along with male sex and younger age, lower scores on generic and condition-specific HrQoL were significantly associated with more externalizing problems. There was also a significant interaction effect between condition-specific HrQoL and treatment status, though its contribution to explain additional variance was non-significant.

**Table 6:** Main and interaction effects of clinical and psychosocial variables on patient-reported externalizing problems.

	Patient-reported externalizing problems							
	Covariates		Clinical variables main effects		Psychosocial variables main effects		Interaction effects	
	Δ*R*^2^ = .06		Δ*R*^2^ = .003		Δ*R*^2^ = .16		Δ*R*^2^ = .02	
	Δ*F*_(2, 213)_ = 6.61^**^		Δ*F*_(2, 211)_ = 0.31		Δ*F*_(4, 207)_ = 10.66^**^		Δ*F*_(4, 203)_ = 1.31	
	β	*t*	β	*t*	β	*t*	β	*t*
Children’s sex ^a^	.20	2.97^**^	.20	2.96^**^	.22	3.45^**^	.24	3.70^**^
Children’s age	-.15	-2.30^*^	-.15	-2.23^*^	-.15	-2.42^*^	-.17	-2.64^**^
Treatment status ^b^			-.03	-0.37	-.14	-2.07^*^	-.11	-1.48
Current height deviation ^c^			.06	0.78	-.02	-0.28	-.02	-0.22
Generic QoL					-.24	-3.34^**^	-.34	-3.22^**^
Specific QoL					-.26	-2.61^**^	-.08	-0.60
Coping					-.08	-1.15	-.10	-1.23
Beliefs					-.004	-0.04	-.04	-0.37
Generic QoL * Treatment							.11	1.17
Specific QoL * Treatment							-.22	-2.20^*^
Coping * Height deviation							.02	0.23
Beliefs * Height deviation							.04	0.40
Model summary	*R*^2^ = .06		*R*^2^ = .06		*R*^2^ = .22		*R*^2^ = .24	
	*F*_(2, 213)_ = 6.61^**^		*F*_(4, 211)_ = 3.44^**^		*F*_(8, 207)_ = 7.37^**^		*F*_(12, 203)_ = 5.38^**^	

^a^ Children’s sex: 0 –female, 1 –male

^b^ Treatment status: 0 –treated; 1 –untreated

^c^ Current height deviation: 0 –achieved normal height (> -2sd), 1 –current short stature (≤ -2sd).

^*^ *p* ≤ .05

^**^ *p* ≤ .01, two-tailed.

**Table 7:** Main and interaction effects of clinical and psychosocial variables on parent-reported externalizing problems.

	Parent-reported externalizing problems							
	Covariates		Clinical variables main effects		Psychosocial variables main effects		Interaction effects	
	Δ*R*^2^ = .13		Δ*R*^2^ = .02		Δ*R*^2^ = .11		Δ*R*^2^ = .01	
	Δ*F*_(2, 215)_ = 15.82^**^		Δ*F*_(2, 213)_ = 2.18		Δ*F*_(6, 207)_ = 4.88^**^		Δ*F*_(7, 200)_ = 0.49	
	β	*t*	β	*t*	β	*t*	β	*t*
Children’s sex ^a^	.22	3.51^**^	.23	3.56^**^	.18	2.86^**^	.17	2.47^*^
Children’s age	-.29	-4.51^**^	-.29	-4.59^**^	-.30	-4.65^**^	-.30	-4.53^**^
Treatment status ^b^			-.13	-1.92	-.15	-2.25^*^	-.15	-2.06^*^
Current height deviation ^c^			-.01	-0.10	-.06	-0.76	-.06	-0.80
Generic QoL					-.30	-4.39^**^	-.31	-4.38^**^
Specific QoL					.07	0.61	.11	0.62
Coping					.07	1.07	.08	0.98
Beliefs					-.11	-1.23	-.12	-0.89
Future					-.01	-0.07	-.08	-0.44
Effects on parents					-.08	-0.85	-.08	-0.55
Specific QoL * Treatment							.11	0.77
Future * Treatment							-.20	-1.25
Specific QoL * Height deviation							-.15	-0.95
Coping * Height deviation							-.01	-0.15
Beliefs * Height deviation							.002	0.02
Future * Height deviation							.28	1.52
Effects on parents * Height deviation							-.01	-0.05
Model summary	*R*^2^ = .13		*R*^2^ = .15		*R*^2^ = .25		*R*^2^ = .26	
	*F*_(2, 215)_ = 15.82^**^		*F*_(4, 213)_ = 9.08^**^		*F*_(10, 207)_ = 6.96^**^		*F*_(17, 200)_ = 4.23^**^	

^a^ Children’s sex: 0 –female, 1 –male

^b^ Treatment status: 0 –treated; 1 –untreated

^c^ Current height deviation: 0 –achieved normal height (> -2sd), 1 –current short stature (≤ -2sd).

^*^ *p* ≤ .05

^**^ *p* ≤ .01, two-tailed.

A similar pattern of socio-demographic and psychosocial predictors emerged with parent-reported externalizing problems (Table 7), explaining 13% and 11% of its variance. Parent-reported externalizing problems were significantly associated with male sex, younger age and lower generic HrQoL. Clinical variables or interaction effects between clinical and psychosocial variables were unrelated to parent-reported externalizing problems.

## Discussion

To our knowledge, this is the first study to examine both HrQoL and psychological problems conjointly as indicators of psychosocial adaptation in short statured youth. We identified a greater risk for internalizing problems and impaired HrQoL in children with current short stature as compared to children who had achieved normal height. We also identified HrQoL questionnaires as screening tools for detecting and referring patients with special needs in terms of psychological assessment and intervention. These results are based on a large sample of European short statured patients and a multi-informant approach to pediatric outcomes assessment by incorporating both patient- and parent-reports of HrQoL and psychological problems.

While the frequency distribution of patient-reported psychological problems is equivalent to population norms, it is noteworthy that twice as many short statured children (19.7%) compared to norm populations were assessed within the SDQ’s abnormal range by their parents. The greater likelihood for parents to report more psychological problems than children themselves has been described in previous research on pediatric chronic conditions and suggests that parents may be more aware of their children’s current and future functional limitations [66, 67]. In addition, patients with current short stature presented lower condition-specific HrQoL than their peers with achieved normal height, and untreated patients had lower generic HrQoL than those who are/were receiving GH treatment. Such HrQoL impairments in both generic and condition-specific levels indicate, that children with short stature may face numerous distressing issues that are common to other chronic conditions (e.g., restrictions on gratifying activities), as well as some that are specifically related to their height (e.g., juvenalization).

A second set of results showed that children’s internalizing and externalizing problems were better explained by HrQoL than by height-related factors, both from the patients’ and parents’ perspectives. These results are consistent with the premise that internalizing and externalizing problems in short statured children have been inappropriately attributed exclusively to their height [3]. Still, parents of children with current short stature reported significantly more internalizing problems than parents of children with achieved normal height. This is also supported in a previous study that showed a significant impact of current height deviation on coping behaviors and HrQoL of children/adolescents with short stature [68], suggesting that effects of height deviation on psychological problems could be indirect, via lower HrQoL scores. In addition, a significant proportion of the variance in patient-reported internalizing problems was explained by interaction effects between HrQoL and treatment status. Patients who are/were receiving GH treatment tend to present fewer internalizing problems as their generic HrQoL increases. Conversely, the negative association between condition-specific HrQoL and internalizing problems was stronger for the untreated group. The absence of significant associations between better condition-specific HrQoL and fewer internalizing symptoms for the GH treated group suggests that other factors such as the burden of daily treatments, which includes the need of daily self-injections and frequent medical appointments, may also impact on children’s internalizing problems [37]. However, the generic KIDSCREEN questionnaire may have underestimated the treatment-related burden, while the condition-specific QoLISSY questionnaire was more sensitive in detecting subtle but clinically significant impairments on the basis of treatment burden factors. Such differences in instrument sensitivity between generic and targeted measures have been reported before [69].

Moreover, the effects of the child’s short stature on parents themselves influenced the parents’ perceptions of their children’s internalizing problems. Similar results were reported in other chronic health conditions. White-Koning and colleagues (2007) described a significant association between higher levels of parenting stress and parents’ underrating of HrQoL of their children with cerebral palsy, and Annett, Bender, DuHamel, and Lapidus (2003) reported a significant negative link between illness-related burden experienced by parents and their perceptions of HrQoL in children with asthma [70, 71]. Beyond assuming biases, these results may reflect the impact of parental behavior on their children’s psychosocial functioning [72]. Differences between patient and parent views might also reflect the life long experience of being short for children, while parents maybe more prone to social comparison processes regarding the height of their children [73].

Finally, male sex and younger age emerged as significantly associated with more externalizing problems, both patient- and parent-rated. Numerous risk factors for poor adaptation to short stature have been described in the literature [74], many of which also apply. Nevertheless, social expectations and stereotypes associated with the male gender may influence children’s self-esteem and behavior in that it is socially more acceptable to be short as a girl as compared to a boy [20].

### Limitations of the Study

The cross-sectional design of the study, which precludes the inference of causality, should be considered when interpreting these results. Due to its cross-sectional design, the positive effects of GH-induced growth and HrQoL improvement in psychological problems over time could not be assessed in the present study. Also the hypothesis of reciprocal associations between HrQoL and psychological problems should be understood and appreciated. Because psychosocial adaptation to chronic health conditions is an ongoing dynamic process, rather than a static outcome, further longitudinal studies are needed to examine the effects of achieved height and treatments over time.

A second limitation relates to the generalizability of results. Although we intended to recruit primary caregivers regardless of their sex, our sample was mainly composed of mothers. This disproportionate participation of mothers and fathers is common in pediatric research [75]. However, fathers’ involvement in parental caregiving has been related to fewer psychological problems in their children [76, 77] and further research should examine the role of caregivers’ sex in the psychosocial adaptation of short statured youth. Third, differences in psychosocial adaptation outcomes between children/adolescents with GHD and ISS could not be examined because few ISS patients were treated in the target countries included in the study due to regulation of rhGH treatment [17]. Finally, social and cultural environment play an important role in adaptation to short stature [74]. Although the cross-cultural equivalence of the patient–and parent-reported QoLISSY questionnaire has been described in a previous paper [78], due to the limited number of participating families in some countries such cultural aspects could not be addressed in this study.

### Implications for Clinical Practice and Research

This study uses a novel analytical approach to provide a better understanding of the psychosocial consequences of GHD and ISS and may help scientists and clinicians to identify vulnerable groups of pediatric patients that could benefit from further assessment and referral. Clinicians should be aware that short statured children, particularly those with current height below -2SD from the norms, are at increased risk for internalizing problems. The incorporation of routine HrQoL assessment in pediatric healthcare may contribute to a better understanding of the disease burden, help examine treatment outcomes, evaluate quality of care and assist in tailoring interventions according to patients’ needs [45]. Screening measures to be used in pediatric medical settings should be brief, whilst presenting good reliability and validity; be easily completed by patients/parents and easily scored and interpreted by clinicians; and be responsive to significant differences or changes in patients’ health status [44]. Both, the KIDSCREEN and the QoLISSY questionnaires meet these general requirements and are also available in short forms, thus providing a more economic and less time-consuming approach to HrQoL assessment [79, 80]. Considering the negative associations between HrQoL and internalizing symptoms, children with low HrQoL scores, particularly untreated patients, should be referred for specialized assessment. Psychological interventions in pediatric short stature are particularly important, given the key role of psychological functioning in adherence to prescribed treatments, which in turn has implications for their health, as well as for healthcare resource utilization [32].

In addition to include assessment of the patients’ perspectives, parent-reports of HrQoL and psychological problems should be considered in clinical practice, because parents assume an important role in health care use and treatment compliance and they can be more sensitive in detecting minor but clinically significant changes in their child’s behavior. Moreover, pediatric short stature and GH treatments also have significant effects on parents, who may exhibit psychosocial difficulties affecting their health and wellbeing. Because children’s and parents’ adaptation outcomes are intrinsically related [72], pediatricians should consider both, the child and the family as patients, and provide screening and appropriate referral for parents’ presenting maladaptive height-related beliefs, concerns and psychosocial functioning.
